# Investigating interactions between types of order in categorization

**DOI:** 10.1038/s41598-022-25776-0

**Published:** 2022-12-14

**Authors:** Giulia Mezzadri, Patricia Reynaud-Bouret, Thomas Laloë, Fabien Mathy

**Affiliations:** 1 Cognition and Decision Lab, Columbia University, New York City, 10027 USA; 2 Laboratoire J.A. Dieudonné UMR CNRS 7351, Université Côte d’Azur, Nice, 06108 France; 3 Laboratoire Bases, Corpus, Langage UMR CNRS 7320, Université Côte d’Azur, Nice, 06357 France

**Keywords:** Psychology, Human behaviour

## Abstract

This study simultaneously manipulates within-category (rule-based vs. similarity-based), between-category (blocked vs. interleaved), and across-blocks (constant vs. variable) orders to investigate how different types of presentation order interact with one another. With regard to within-category orders, stimuli were presented either in a “rule plus exceptions” fashion (in the rule-based order) or by maximizing the similarity between contiguous examples (in the similarity-based order). As for the between-category manipulation, categories were either blocked (in the blocked order) or alternated (in the interleaved order). Finally, the sequence of stimuli was either repeated (in the constant order) or varied (in the variable order) across blocks. This research offers a novel approach through both an individual and concurrent analysis of the studied factors, with the investigation of across-blocks manipulations being unprecedented. We found a significant interaction between within-category and across-blocks orders, as well as between between-category and across-blocks orders. In particular, the combination similarity-based + variable orders was the most detrimental, whereas the combination blocked + constant was the most beneficial. We also found a main effect of across-blocks manipulation, with faster learning in the constant order as compared to the variable one. With regard to the classification of novel stimuli, learners in the rule-based and interleaved orders showed generalization patterns that were more consistent with a specific rule-based strategy, as compared to learners in the similarity-based and blocked orders, respectively. This study shows that different types of order can interact in a subtle fashion and thus should not be considered in isolation.

## Introduction

What is the best way to memorize lists of vocabulary in another language? Would you study words category by category (for instance, red–blue–yellow–etc., then dog–cat–canary–etc.) to reinforce associations within categories, or would you rather alternate words from different categories (for instance, black–dog–yellow–canary–white–cat–etc.) because you think the two categories can better benefit each other when intertwined? Also, how would you arrange words within a category? Would you first learn words that are phonetically similar (for instance, cat–bat–etc.) or words that are related by a given structure (for instance, warm vs. cold colors) to induce sub-groups, or would you rather study them in random order to facilitate more personal associations from the learner? We believe that these alternative sequences and their combination inevitably produce different outcomes.

A few studies have shown that presentation order influences learning speed and retention in a variety of domains such as memory^1,2^, eyewitness identification^3^, serial recall^4^, risk perception^5,6^, and categorization^7–12^. In categorization for instance, considerable effort has been directed toward the study of between-category orders^13–27^. More specifically, between-category orders have been thoroughly examined by manipulating interleaving (in which categories are presented alternatively, i.e. a Category-1 member followed by a Category-2 member) versus blocking (in which members of a single category are presented in a row on successive trials, i.e. a Category-1 member followed by other Category-1 members). In addition to a spacing effect^28–31^, interleaving stimuli of different categories has been shown to highlight the differences between these stimuli, thus facilitating learning and transfer^18,25,27,32–34^. However, there has also been evidence in favor of blocking members of a same category^29,35–38^.

A lesser number of studies have focused on within-category order effects on category learning^39,40^. Originally explored in word recall^41^ and old-new recognition tasks^42^, the manipulation of order within members of a same category has moderately been extended to categorization tasks after the original work of Elio^43–46^. An example of within-category manipulation is the similarity-based order in which stimuli of a same category are arranged in order to maximize the similarity between contiguous examples. This typical manipulation has been contrasted with a rule-based order in which stimuli obeying a rule precede the exceptions to the rule. For instance, Mathy and Feldman^44^ have found that presenting stimuli in a “rule plus exceptions” ordering facilitates learning as compared to both similarity-based and dissimilarity-based orders. The study of rule-based versus similarity-based order is particularly relevant since these order manipulations match two extreme ways of learning: an inductive process based on abstraction, and an elementary process based on associative mechanisms^47^. The rule-based order is supposed to help participants abstract the logical rule describing the stimuli, while the similarity-based order uses temporal proximity to strengthen the memory traces of contiguous items.

The rationale for the present study is to investigate the effect of combinations of presentation orders on memorization and subsequent categorization. While all above-mentioned studies have manipulated a single factor (either between-category or within-category orders), the present study simultaneously manipulates between-category (blocked vs. interleaved), within-category (rule-based vs. similarity-based), and across-blocks orders (constant vs. variable). Table 1 illustrates how these three manipulations were combined. Because we used generic types of within-category orders (rule-based vs. similarity-based), the order in which stimuli were presented could still vary once this type of order was chosen. For instance, if we presented a series of faces to be categorized by grouping them by gender (or by hair color), we could still apply variations within faces of the same gender (or hair color).

**Table 1:** Combination of the three manipulations (within-category, between-category, and across-blocks) that generates the eight conditions of the experiment. The last column offers an example of a two-block sequence for each condition. Stimuli can belong to either Category *A* (letters *a*, $a'$, *A*, and $A'$) or Category *B* (letters *b*, $b'$, *B*, and $B'$). These categories were chosen for this table as simple as possible to exemplify different types of order more easily. Note that this category structure is not the one used in our experiment. Stimuli associated with the main rule are in capital letters, while the exceptions are in small letters.

Index	Within-category	Between-category	Across-blocks	Two-block sequence
1st	Rule-based	Blocked	Constant	$AA'aa'BB'bb'$–$AA'aa'BB'bb'$
2nd	Rule-based	Blocked	Variable	$AA'aa'BB'bb'$–$A'Aaa'BB'b'b$
3rd	Rule-based	Interleaved	Constant	$ABA'B'aba'b'$ –$ABA'B'aba'b'$
4th	Rule-based	Interleaved	Variable	$ABA'B'aba'b'$–$A'B'ABa'bab'$
5th	Sim.-based	Blocked	Constant	$aa'A'Abb'B'B$ –$aa'A'Abb'B'B$
6th	Sim.-based	Blocked	Variable	$aa'A'Abb'B'B$ –$a'aAA'b'bBB'$
7th	Sim.-based	Interleaved	Constant	$aBa'B'A'b'Ab$ –$aBa'B'A'b'Ab$
8th	Sim.-based	Interleaved	Variable	$aBa'B'A'b'Ab$ –$Ba'B'A'b'Aba$

This variability in the choice of the sequence to be presented allowed us to consider a novel manipulation, that we called across-blocks manipulation in our design. For instance, an example of across-blocks manipulation would consist of varying the order of the faces within the same gender (or hair color) from one block to another. Manipulations across-blocks were thought to be particularly important because they have not been explicitly addressed by previous studies. Studies involving between-category orders have generally randomized the order of stimuli from one block to another, whereas the past literature on within-category orders has essentially focused on two extreme ways of presenting stimuli. In^44^, the authors chose to adopt variable orders across blocks while interspersing the stimuli. Conversely, in^45^ a single fully-blocked sequence was generated for each participant and used across all blocks. Although in both cases the “rule plus exceptions” ordering yielded better learning, we still do not know whether the rule-based order could be more, less, or equally affected by such manipulations as compared to the similarity-based order. We thus thought that the effect of across-blocks orders should be better comprehended using a more exhaustive manipulation of the way the stimuli are presented.

With regard to the interactions between factors manipulating order, we expected an interaction between between-category and across-blocks orders. We know from the literature that repeating the same sequences using a Hebb-repetition learning^48,49^ increases memorization. We therefore hypothesized that blocking constant orders would offer the largest memory gain, regardless of within-presentation orders (that is, regardless of whether presentation induces memorization of stimuli or memorization of rules). Constant orders should favor both associations by similarity or clustering by rules. Blocking should also reinforce the formation of one of the two categories, in particular when a rule can be extracted for one category.

This is why we also hypothesized a full interaction between our three factors. We expected a maximal beneficial effect using a rule-based presentation order for which the categories would be blocked and using constant orders across blocks. Because grouping processes should greatly benefit the extraction of rules, this combined condition should let participants more easily extract rules and exceptions (in particular because we targeted a category structure that is especially prone to induce the formation of rules, as explained below). On a more theoretical level, the presence or absence of interactions should be informative for current categorization models, which in the future could be adapted to account for different order combinations. On a more practical level, our findings could have a huge impact on how to organize learning (for instance in the classroom), train experts (for medical image classification), or improve machine learning algorithms.

Because we tested the effect of new factors manipulating presentation orders and combinations of these factors, we decided to use the widely employed 5–4 category structure from Medin and Schaffer^50^ to generate the stimuli and categories, and to study the strategies engaged by participants. A detailed description of the 5–4 category set can be found in Sect. 2.2. This structure has been analyzed in numerous studies and has influenced research in category learning for more than a quarter century^51–66^. Moreover, the artificial structure of this category set allows for the presence of stimuli without a category label. The advantage is that on these stimuli different classification strategies lead to distinctive response patterns, allowing us to study the mental representation of the categories (see details in Sect. 3.2). For these reasons, the 5–4 category set appeared to be a fruitful starting point for our investigation.

## Method

### Participants

Two hundred and sixteen participants contributed to this study. We initially recruited 218 participants, but two participants were excluded from the data set for failing to follow instructions. Among the 216 participants, 130 were sophomore or junior students from University Côte d’Azur who received course credits in exchange for their participation. The remaining 86 participants were recruited on campus or by email on a voluntary basis. We used G$^*$Power^67^ to estimate the power of detecting a small-medium effect size ($f=0.2$) for the interaction between the three types of order manipulation ($2 \times 2 \times 2 = 8$ between-subject groups) with a three-way ANCOVA model, considering 216 participants, 1 co-variate (i.e., the block number), and $\alpha =0.05$. The power achieved was 83%. Note that the data-set corresponding to the first 130 participants has already been used in^68^ for testing categorization models. The experimental procedure was approved by the local ethics committee (CERNI #2020-74) of Université Côte d’Azur and the experiment was performed in accordance with relevant guidelines and regulations. Informed consent was obtained from all participants prior to participation.

### Categories

**Figure 1:**
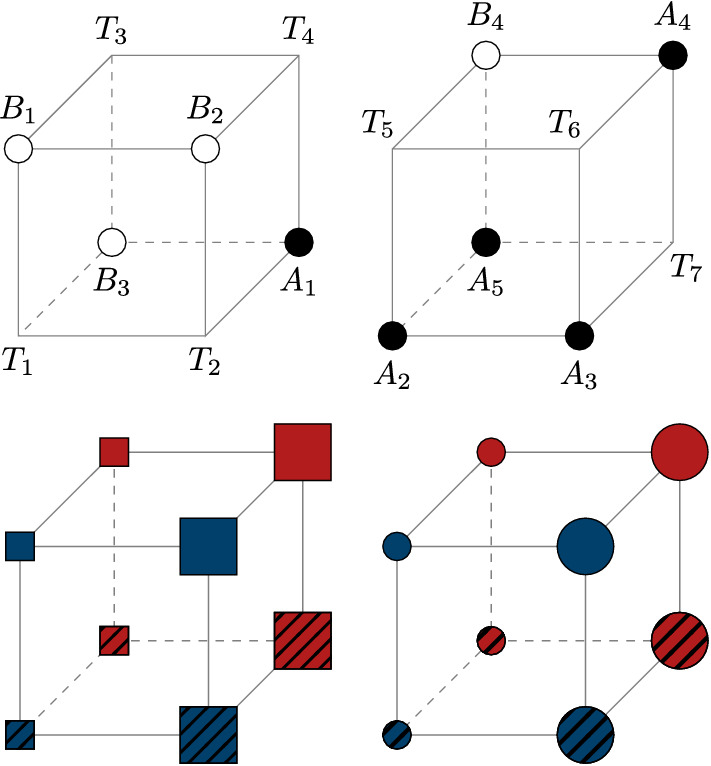
Categories and stimulus items of the categorization task. At the top, the 5–4 category set of Medin and Schaffer^50^, represented here in a Hasse Diagram forming a hypercube. Members of category *A* are represented by black dots, members of category *B* are represented by white dots, and transfer items are represented by empty vertices. At the bottom, illustration of the stimulus items that varied along four Boolean dimensions (Color, Shape, Size and Filling pattern).

Each participant was administrated a single 5–4 category set^50^. This structure is composed of 16 stimuli, varying on four different binary-valued dimensions (see Fig. 1, on the top). In this category set, five stimuli belong to category *A*, four belong to category *B*, and the remaining seven are transfer stimuli. These categories are more structured than random (i.e., a clear rule-plus-exceptions pattern emerges) and are linearly separable. The $5+4=9$ stimuli characterized by a category label were presented in both the learning and transfer phases, whereas the 7 transfer stimuli were presented in the transfer phase exclusively.

### Stimuli

Stimuli varied along four Boolean dimensions (Color, Shape, Size, and Filling pattern). The colors were either blue or red; shapes were either square or circle; sizes were either small or big, and filling patterns were either plain or striped. The combination of these options formed $2^4=16$ items (see Fig. 1, on the bottom). Color distinguished the objects at the front of the hypercube from those at the back, Shape distinguished the objects in the left cube from those in the right cube, Size distinguished the right and left objects within the cubes, and Filling pattern distinguished the objects at the top of the hypercube from those at the bottom. Each dimension was instantiated by the same physical features and the same category structure was applied to these features across participants.

### Phases

A learning phase in which participants were instructed to learn the classification of $5+4=9$ learning stimuli was followed by a transfer phase in which participants were tested upon presentation of 7 novel stimuli (plus the 9 stimuli previously acquired). In the learning phase, both feedback and no-feedback training were used. In particular, two blocks of feedback training (in which the order of the stimuli was manipulated) were followed by one block of no-feedback training (in which stimuli were randomly presented). This pattern was repeated until the end of the learning phase. Each training block (feedback and no-feedback) included nine trials, one for each stimulus. The use of random blocks with no-feedback allowed us to assess learning with neither order manipulation nor feedback interfering with the measure of performance. The unbalanced ratio of two blocks of feedback training followed by one block of no-feedback training aimed at increasing the influence of our manipulation, with the idea that the random block could still interfere with the learning process. Participants had to correctly classify stimuli in three no-feedback blocks of $5+4=9$ stimuli (not necessarily consecutive) to complete the learning phase. The choice of three is arbitrary, but appeared to be a good trade-off between maximizing the memorization of the categories and minimizing the duration of the task (a fundamental point considering that the task was conducted online). Participants were given 200 blocks at the most to reach the learning criterion. Once participants met the learning criterion, the transfer phase was initiated. Participants were informed that they successfully completed the learning phase and that the transfer phase was about to start. The transfer phase was composed of five blocks of 16 stimuli (the $5+4=9$ learning stimuli and the 7 novel stimuli), summing to 80 trials.

### Ordering of stimuli

The experiment was characterized by a full factorial design. Three factors were used, each one having two levels: a within-category order manipulation (Rule-based vs. Similarity-based), a between-category order manipulation (Blocked vs. Interleaved), and a manipulation of order across blocks (Variable vs. Constant). The combination of these factors formed eight conditions (e.g., “Rule-based + Interleaved + Constant”, etc.). For simplicity purposes, each condition is denoted using the first letter of each type of order. For instance, condition “Rule-based + Interleaved + Constant” is denoted R+I+C. As mentioned above, order was only manipulated in the blocks of the learning phase where feedback was provided. The number of participants assigned to each condition is given in Table 2.

**Table 2:** Number of participants assigned to each of the eight conditions of the experiment. The table also includes the order manipulation for the two participants (assigned to conditions R+B+C and R+B+V) who were excluded from the study for not following instructions.

	Rule-based		Similarity-based	
	Constant	Variable	Constant	Variable
Blocked	28	28	27	27
Interleaved	27	27	27	27

#### Within-category order manipulation

In the rule-based order, stimuli were ordered following a “principal rule plus exceptions” structure, meaning that examples obeying the principal rule were presented strictly before the exceptions. The specific “principal rule plus exceptions” structure of our experiment was the following: all striped items belong to category *A* except for the small red square, while all plain items belong to category *B* except for the big red circle (see Fig. 1). Therefore, items $A_1,\,A_2,\,A_3,\,A_5$ were strictly presented before item $A_4$, and items $B_1,\,B_2,\,B_4$ were strictly presented before item $B_3$. The items belonging to the principal rule (whether belonging to categories *A* or *B*) were randomly selected. Presenting stimuli belonging to the dominant rule in a random order was thought to favor an abstraction process, given that other sequences would have increased the risk of temporarily inducing less informative rules, thus delaying learning. Note that instead of using a principal rule based on Filling pattern (plain vs. striped stimuli), we could have used a principal rule based on Shape (circles vs. squares). Indeed, both rules minimize the number of exceptions.

In the similarity-based order, members within a category were presented in a way that maximized the similarity between adjacent learning stimuli. The first stimulus was randomly chosen while subsequent stimuli were (randomly) chosen among those that were the most similar to the immediately previous item. Similarity between two items *x* and *y* was computed by counting the number of common features they shared:

$$\begin{aligned} s_{xy}=\sum \limits _{i=1}^4\mathbbm {1}_{\{x_i=y_i\}}, \end{aligned}$$

where $x_i$ and $y_i$ are the feature values of stimuli *x* and *y* on dimension *i*. For instance, the small plain blue circle and the small striped red square have one single feature in common (small), thus their similarity is 1.

#### Between-category order manipulation

In the blocked study, categories were strictly blocked (*AAAABBBB* or *BBBBAAAA*), while in the interleaved study categories were strictly alternated (*ABABABAB*). Because of the regularity of both patterns, the introduction of random blocks during learning was necessary. Indeed because of these repetitive patterns, participants could have guessed the correct classification without paying attention to the stimuli. The ratio between blocked (or interleaved) blocks and random blocks is 1:3, as for the feedback/no-feedback blocks. Therefore, a random block with no-feedback always follows two blocks in which categories are blocked (or interleaved) and feedback is provided. Note that in random blocks feedback was never provided, whereas in blocked/interleaved blocks feedback was always provided.

#### Across-blocks order manipulation

In the constant manipulation across blocks, the same sequence of stimuli (but obeying the constraints of the between- and within-category orders) was presented in all feedback blocks, while in the variable manipulation across blocks the sequence of stimuli varied from one feedback block to another (again, obeying the constraints of the between- and within-category orders).

### Procedure

The categorization task was computer-driven and was conducted online. Participants received instructions before the task began. In both phases, stimuli were presented one at a time for 3 s on the center of the computer screen. Category *A* was associated with the up key, while category *B* was associated with the down key. Participants had to classify the stimulus in one of the two categories (*A* and *B*) using these two response keys. Once the key pressed, a feedback indicating the correctness of participants’ classification appeared for 1 s at the bottom of the screen (this was the case only in blocks where feedback was provided). If no key was pressed, the text ’too late’ appeared for 1 s at the bottom of the screen. In order to encourage learning, a percentage of correct responses was calculated at the end of each no-feedback block, based on performance on the last no-feedback block only. This percentage was displayed for 1 s right after each no-feedback block.

## Results

### Learning phase

Two of our main questions of interest are *(i)* whether the across-blocks manipulation (constant vs. variable) affects the speed at which the concept is learned, and *(ii)* whether there are interactions between the types of order we manipulated. To answer these questions, we analyzed the time needed by participants to complete the learning phase as a function of the type of order (in Sect. 3.1.1), and we performed a three-way ANCOVA with and without interactions (in Sect.3.1.2) Two additional analyses can be found in Supplementary material A and B. The first analysis examines the number of individuals who did not reach the learning criterion, and shows no significant difference across types of order. The second analysis examines the percentage of correct responses given by participants over the course of the learning phase, and finds faster learning curves in the rule-based order than in the similarity-based order. None of the 216 participants were excluded from the analyses of the learning phase.

#### Analysis of the learning times

**Figure 2:**
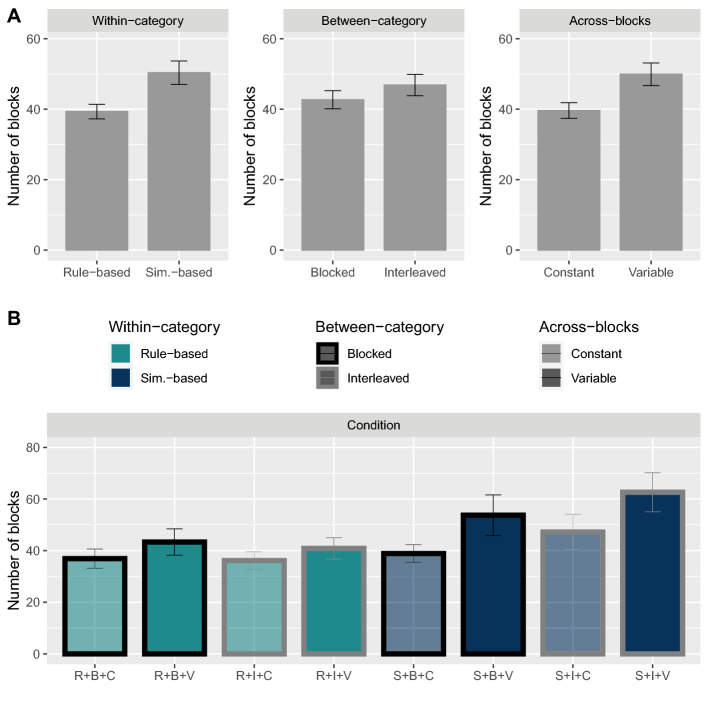
Average number of blocks taken by participants to meet the learning criterion as a function of the experimental conditions, taken separately (**A**) and combined (**B**). In (**B**), color distinguishes the within-category order (light blue for rule-based and dark blue for similarity-based), contrast of borders distinguishes the between-category order (black for blocked and gray for interleaved), and opacity of the colors distinguishes the across-blocks order (semi-transparent for constant and opaque for variable). Error bars show $\pm 1 SE$. Note that only the participants that reached the learning criterion are plotted in this graph.

Figure 2 shows the average number of blocks which were required for participants to meet the learning criterion as a function of the experimental conditions, taken separately (Fig. 2A) and combined (Fig. 2B). Visually, the rule-based order appears more beneficial than the similarity-based order, the blocked order appears somewhat more beneficial than the interleaved one, and the constant condition appears more beneficial than the variable condition. Note that only participants who reached the learning criterion (amounting to 198) were plotted in Fig. 2. To determine which condition led to the fastest learning while accounting for “unsuccessful participants” (i.e., individuals who did not meet the learning criterion), we used two survival analysis techniques: the Kaplan-Meier survival curves and the Cox proportional-hazards model.

**Figure 3:**
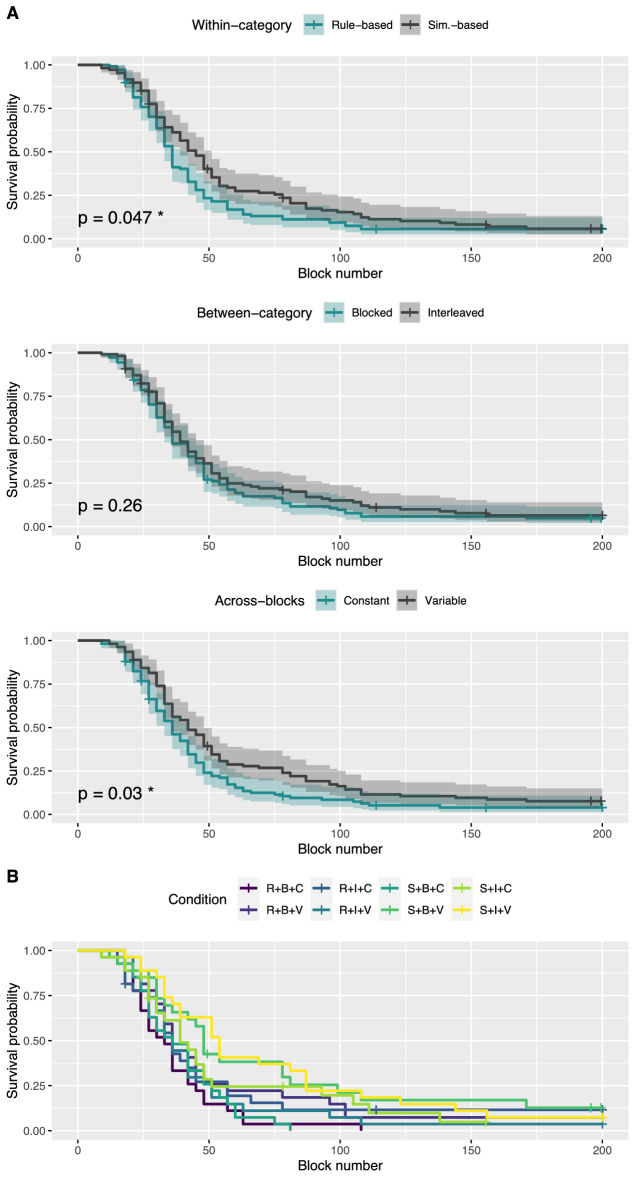
Kaplan-Meier survival curves as a function of block number for each type of order, taken separately (**A**) and combined (**B**). Transparent areas represent the 95% confidence intervals. Significance values of the log-rank test assessing the difference between survival curves are showed on the bottom-left side of each graph of (**A**). Significance values were corrected for multiple comparisons using the Benjamini-Hochberg correction with a FDR $\le .05$.

*Kaplan-Meier survival curves.* We used the Kaplan-Meier estimator^69^ to estimate the expected duration of time until the successful completion of the learning phase, considering data from participants who did not complete the task as censored. Figure 3 shows the survival probability as a function of block number for each type of order, taken separately (Fig. 3A) and combined (Fig. 3B). The survival probability estimates how likely participants assigned to a given condition are to continue the task (i.e., to not meet the learning criterion). The log-rank test was performed to evaluate the difference between survival curves, and significance values were corrected for multiple comparisons using the Benjamini-Hochberg method of false discovery rate control (FDR $\le .05$). Note that it is sufficient to compare the adjusted *p*-values to 0.05 to determine if they are significant for a FDR $\le .05$. The adjusted *p*-values were significant for the within-category and across-blocks orders ($p =.047$ for rule-based vs. similarity-based, and $p = .03$ for constant vs. variable), but not for the between-category order ($p=.26$). This shows that learning was faster in the rule-based and constant orders as compared to the similarity-based and variable orders, respectively.

**Figure 4:**
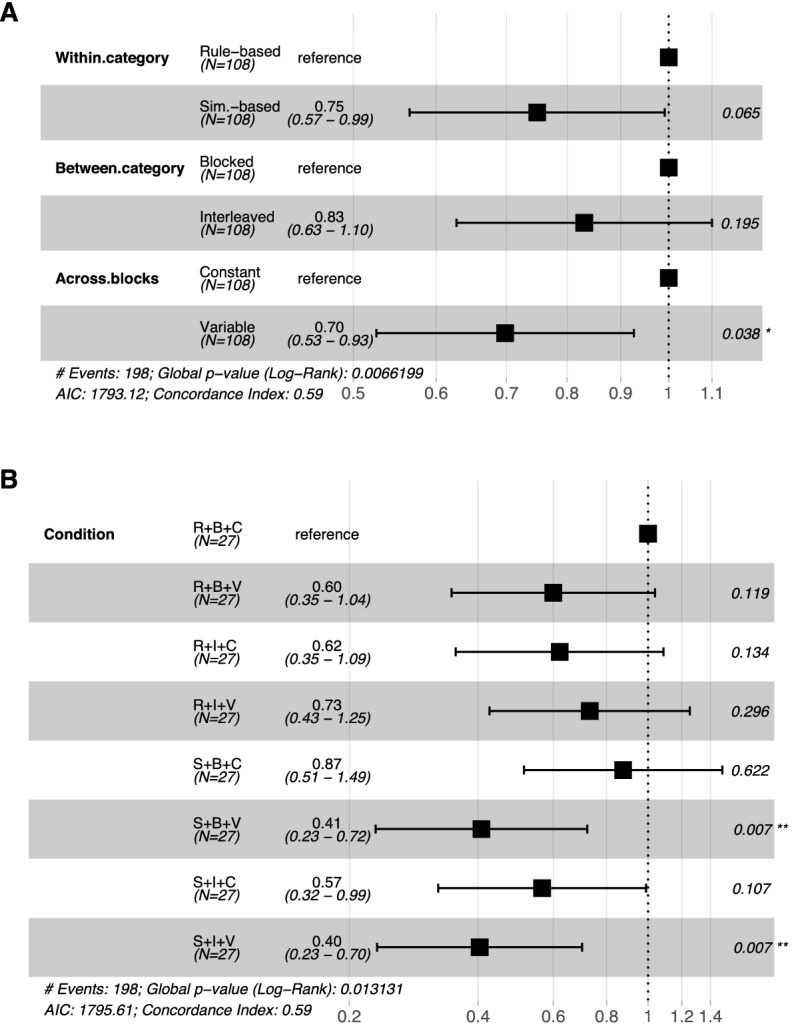
Results of the application of the Cox model as a function of the types of order, taken separately (**A**) and combined (**B**). Hazard ratios and their 95% confidence intervals are showed in the middle. Significance values of the Wald test (showed on the right) were corrected for multiple comparisons using the Benjamini-Hochberg correction with a FDR $\le .05$.

*Cox proportional-hazards model.* Similarly to the Kaplan-Meier estimator, the Cox model^70^ allows us to consider failures to complete the task as censored data, avoiding to remove unsuccessful participants. This model is particularly advantageous because of its ability to simultaneously account for multiple variables. Therefore, we use it to simultaneously analyze the influence of the three types of order (within-category, between-category, across-blocks orders) on survival probability. Figure 4A shows the result of the Cox model as a function of our three variables (within-category, between-category, across-blocks orders). The graphs show that the similarity-based order, the interleaved study, and the variable manipulation across-blocks reduced participants’ hazard ratio as compared to their respective reference condition (i.e., rule-based order, blocked study, and constant manipulation across-blocks). This means that these types of order were found to reduce participants’ speed to meet the learning criterion. However, only the impact of across-blocks manipulations was significant ($p = .065$ for within-category orders, $p = .195$ for between-category orders, and $p =.038$ for across-blocks orders). Figure 4B shows the result of the Cox model as a function of the conditions. Hazard ratio of conditions S+B+V and S+I+V were found to be significantly smaller than the hazard ratio of the reference condition R+B+C ($p = .007$ for both conditions), meaning that participants in conditions S+B+V and S+I+V were statistically slower than participants in condition R+B+C in reaching the learning criterion. The *p*-values for the remaining conditions are reported in Fig. 4B. All the significance values in Fig. 4 were corrected for multiple comparisons using the Benjamini-Hochberg correction with a FDR $\le .05$.

#### Analysis of the interactions between the types of order

With the previous survival analyses, we only investigated main effects and potentially ignored any subtle interaction between the types of order. In order to assess whether the manipulations interact in a nuanced fashion, we performed a three-way ANCOVA ($2\times 2\times 2$ with interactions) with within-category order (rule-based vs. similarity-based), between-category order (blocked vs. interleaved), and across-blocks manipulation (constant vs. variable) as between-subject factors. The number of correct responses per block was the dependent variable and block number was the only co-variate. To ensure an equal contribution from each participant, we completed participants’ responses until block number 63. Since 80% of the participants ended (successfully or not) the learning phase before block number 63, this choice allowed us to ensure an equal number of observations for each participant, while limiting the number of observations that were removed or added. There were 170 participants who ended the learning phase before block number 63. Five of them dropped out of the experiment, while the remaining 165 met the learning criterion. Among the 165 participants who successfully finished the learning phase (and therefore had 100% accuracy in their last no-feedback block), 116 (=70%) made no mistake, 30 (=18%) made one mistake, and 19 (=12%) made more than one mistake in their penultimate no-feedback block. Since the majority of the participants who met the learning criterion reached a stable optimal strategy (88% of them made one or no mistake in the last two no-feedback blocks), it is reasonable to think that they would have continued to perform optimally after completing the learning phase. Therefore, participants’ data were filled in by iterating their last no-feedback block until block number 63. Note that with this fill-in method data from participants who met the learning criterion was completed with 100% accuracy. In Supplementary material C, we implement an alternative way of filling in the data by iterating participants’ average performance across the last two no-feedback blocks. The results of this analysis are qualitatively the same as those described below, with the exception of the interaction between the three types of order which turned out significant when using the alternative fill-in method. A probit transformation was applied to the dependent variable in order to meet the assumption of normality. Twenty observations among the 4536 available were found to be multivariate outliers and were excluded from the analysis (here, by observation we mean the performance obtained by a specific participant in a specific block). Note that the ANCOVA analysis was only run on no-feedback blocks.

Block number was a significant predictor of participants’ performance ($F(1,5877)=1886.13$, $p<.0001$, $\eta _p^2=.30$). After controlling for block number, the main effect of within-category order was significant ($F(1,246)=78.91$, $p<.0001$, $\eta _p^2=.017$), indicating that participants in the rule-based order ($M=2.71$, $SE=.038$) performed better than those in the similarity-based order ($M=2.24$, $SE=.038$). The main effect of between-category order was also significant ($F(1,71)=22.65$, $p<.0001$, $\eta _p^2=.005$), with participants in the blocked order ($M=2.60$, $SE=.038$) showing higher performance than those in the interleaved order ($M=2.35$, $SE=.038$). Finally, the main effect of across-blocks was significant ($F(1,198)=63.39$, $p<.0001$, $\eta _p^2=.014$), with the constant order ($M=2.68$, $SE=.038$) being more beneficial than the variable one ($M=2.27$, $SE=.038$). With regard to the interactions, we found a first interaction between within-category and across-blocks orders ($F(1,61)=19.66$, $p<.0001$, $\eta _p^2=.004$), a second interaction between between-category and across-blocks orders ($F(1,50)=15.96$, $p<.0001$, $\eta _p^2=.004$), and a third interaction between within-category and between-category orders ($F(1,17)=5.46$, $p=.022$, $\eta _p^2=.001$). Finally, the interaction between the three types of order was not significant ($F(1,8)=2.55$, $p=.11$, $\eta _p^2=.0006$). All *p*-values were corrected for multiple comparisons using the Benjamini-Hochberg correction^71^ with a FDR $\le .05$.

**Figure 5:**
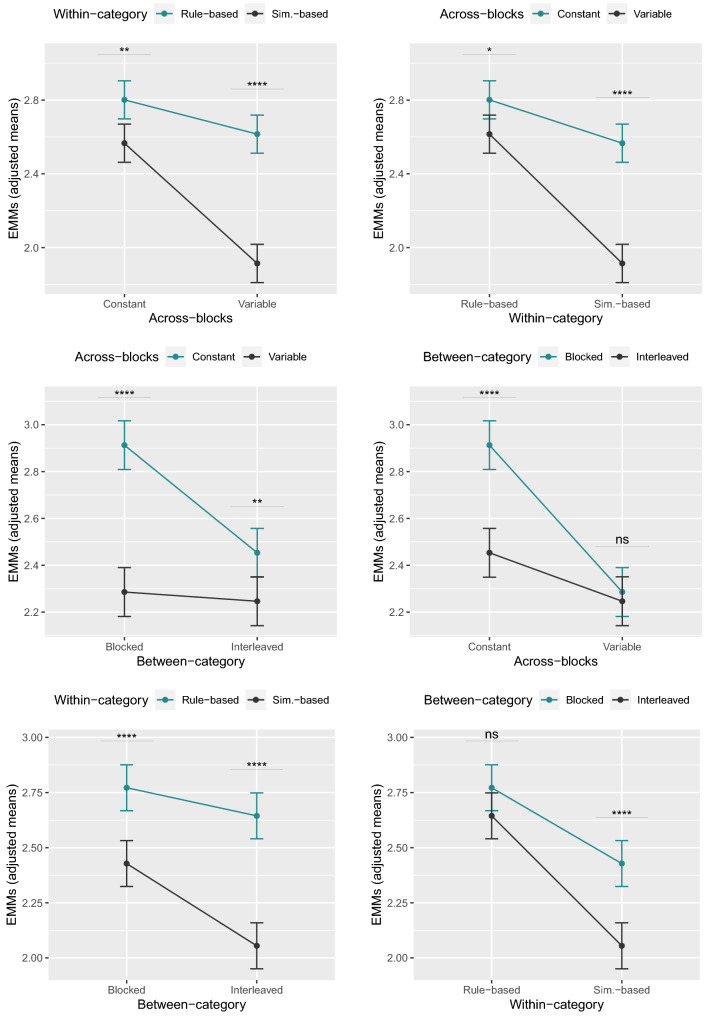
Simple main effect analysis for the significant interactions. Estimated marginal means (EMMs) for the interaction between within-category and across-blocks orders (on the top), between between-category and across-blocks orders (in the middle), and between within-category and between-category orders (on the bottom). Significance values were adjusted using the Benjamini-Hochberg correction with a FDR $\le .05$.

To further investigate the significant interactions, we conducted an analysis of simple main effects applying again the Benjamini-Hochberg correction. The simple main effect of within-category order was significant in both the constant ($p = .002$) and variable ($p < .0001$) across-blocks groups. Similarly, the simple main effect of across-blocks order was significant in both the rule-based ($p=.013$) and similarity-based ($p < .0001$) within-category groups. The simple main effect of across-blocks order was significant in both the blocked ($p < .0001$) and interleaved ($p=.006$) between-category groups, whereas the simple main effect of between-category order was significant in the constant order ($p < .0001$), but not in the variable one ($p=.6$). Finally, the simple main effect of within-category order was significant in both the blocked ($p < .0001$) and interleaved ($p< .0001$) between-category groups, whereas the simple main effect of between-category order was significant in the similarity-based order ($p < .0001$), but not in the rule-based one ($p=.09$). These effects can be visualized in Fig. 5.

To complete the analysis, we assessed the difference in performance between the three-way ANCOVA with and without interactions. The F-test was significant ($p<.0001$), showing that the model with interactions was significantly better than the model without interactions. To assess the robustness of our results, we applied the three-way ANCOVA multiple times by varying the block number until which the responses were completed (i.e., block number 48, 51, 55, and 81 corresponding to the 65%, 70%, 75%, and 85% quantile at which participants ended the learning phase). The results were qualitatively the same, although the interaction between within-category and between-category orders was no longer significant when responses were completed until block number 48 ($p=.1$) and block number 51 ($p=.08$). For consistency purposes, our discussion will only focus on the two interactions that were found significant across every case (i.e., the interaction between within-category and across-blocks orders, and the one between between-category and across-blocks orders).

### Transfer phase

Our next aim was to determine whether the types of order affected *(i)* performance on learning stimuli and *(ii)* generalization patterns on transfer stimuli during the transfer phase. Because we were interested in studying performance and generalization patterns in participants who learned the studied categories, the 18 participants who did not meet the learning criterion were excluded from the analyses. Data from 14 participants were additionally excluded because considered as outliers. Eleven of them pressed the key associated with Category B less than 10% of the trials, and the remaining three pressed no key on more than 15% of the trials. To recap, analyses of the transfer phase were performed on 184 participants. The distribution among the experimental conditions of the participants who were excluded is shown in Table 3.

**Table 3:** Frequency and presentation order of the 32 participants who were excluded from the analyses of the transfer phase. Eighteen participants did not reach the learning criterion, plus 14 participants were excluded because considered as outliers (among the 14 participants, 11 pressed the key associated with Category B less than 10% of the trials, and three participants pressed no key on more than 15% of the trials.

	Rule-based		Similarity-based	
	Constant	Variable	Constant	Variable
Blocked	1	4	5	5
Interleaved	5	1	5	6

#### Analysis of performance on learned stimuli

**Figure 6:**
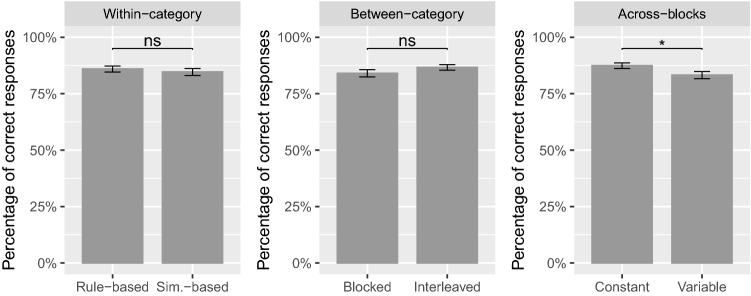
Percentage of correct responses for the learning stimuli presented during transfer, as a function of the types of order (taken separately). Percentage of correct responses was first computed for each participant before being averaged across participants. Error bars show $\pm 1 SE$. Statistical significance of the two-sided Wilcoxon-Mann-Whitney test is shown on the top.

Figure 6 shows the percentage of correct responses for the learning stimuli presented during transfer, as a function of the types of order (taken separately). The percentage of correct responses was first computed for each participant and then averaged across participants. The two-sided Wilcoxon-Mann-Whitney test was performed to assess the difference in performance between the two conditions within each type of manipulation. Only the difference between constant versus variable across-blocks manipulations was significant ($p = .628$ for rule-based vs. similarity-based, $p = .673$ for blocked vs. interleaved, $p = .035$ for constant vs. variable), indicating that participants in the constant condition learned the concept better than those in the variable condition. With regard to the within-category and between-category orders, no evidence was found suggesting that performance on learning stimuli was significantly impacted by the type of order.

**Figure 7:**
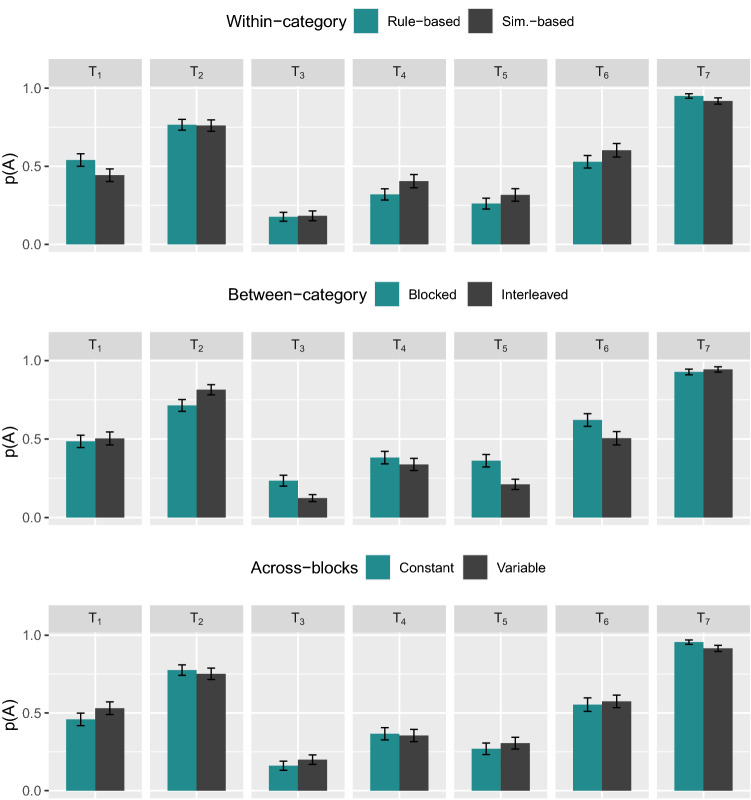
Average classification of the transfer items ($T_1$, $T_2$, $T_3$, $T_4$, $T_5$, $T_6$, $T_7$) during the transfer phase (amounting to five blocks) as a function of types of order (taken separately). Quantity *p*(*A*) is the observed proportion that each of the stimuli labeled under the abscissa was classified into category *A* during the transfer phase. Quantity *p*(*A*) was first computed for each participants before being averaged across participants. Average classification of the nine learning items is not included. Error bars show $\pm 1 SE$.

#### Analysis of generalization patterns on transfer stimuli

Figure 7 shows the average classification of the transfer items over the course of the transfer phase as a function of type of order (taken separately). Quantity *p*(*A*) is the observed proportion that each transfer item was classified into category *A* during transfer. To determine whether participants in different conditions applied different strategies for the classification of novel stimuli, we computed the distance of the observed generalization patterns to four specific strategies (distances were computed using the *L*1 metric and were normalized).

**Figure 8:**
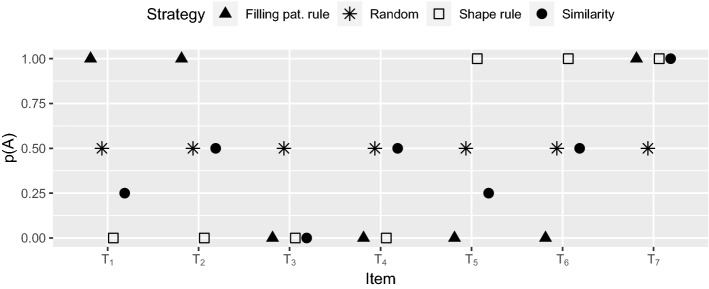
Putative classification of the transfer items ($T_1$, $T_2$, $T_3$, $T_4$, $T_5$, $T_6$, $T_7$) as a function of the applied strategy (rule-based strategy using Filling pattern, rule-based strategy using Shape, similarity-based strategy, and random strategy). Quantity *p*(*A*) is the putative probability for a chosen strategy to classify into category *A* each of the stimuli labeled under the abscissa.

We considered the following strategies: a rule-based strategy that uses Filling pattern (plain vs. striped stimuli) as main rule, a rule-based strategy that uses Shape (circles vs. squares) as main rule, a similarity-based strategy, and a random strategy. Participants adopting a rule-based strategy would classify new stimuli on the basis of the main rule (Filling pattern or Shape, depending on the chosen main rule), whereas participants adopting a similarity-based strategy would classify new stimuli on the basis of their similarity to the closest stored items. In the random strategy, novel stimuli would be randomly classified (50% of chance to classify them into category *A*). The rule-based strategy that uses Shape as main rule was included because Shape (as Filling pattern) allows participants to minimize the number of exceptions when used as the diagnostic dimension (see Sect. 2.5). For simplicity, the two rule-based strategies are called the filling pattern and shape rules, whereas the similarity-based strategy is called similarity strategy. Putative classification of the transfer stimuli for each of the above-mentioned strategies is shown in Fig. 8.

**Figure 9:**
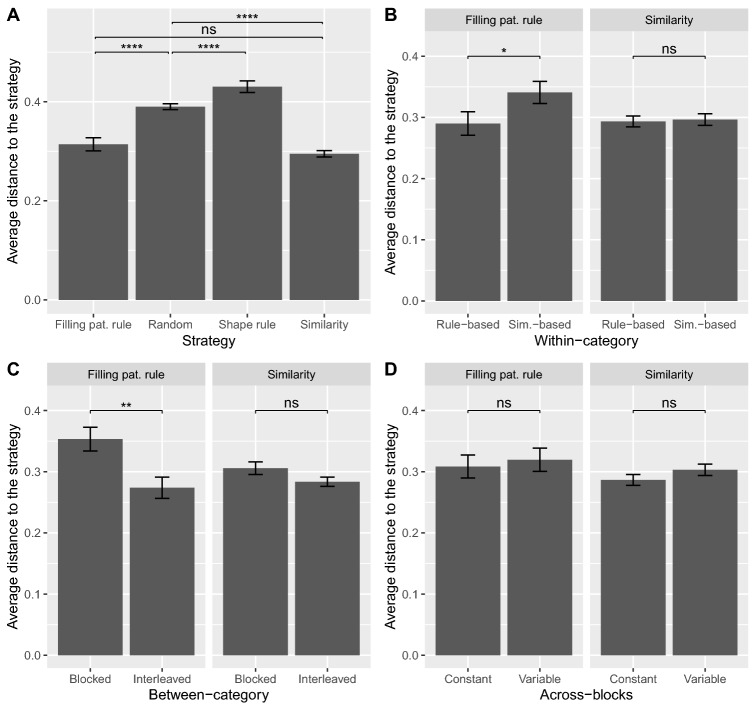
Average distance of participants’ generalization patterns on novel stimuli to specific strategies (rule-based strategy using Filling pattern, rule-based strategy using Shape, similarity-based strategy, and random strategy), for all participants (**A**) and for each type of order within the same manipulation (**B**, **C**, **D**). Distances were first computed for each participant before being averaged. The *L*1 norm was used and distances were normalized prior to averaging. Asterisks show the significance of the two-sided Wilcoxon-Mann-Whitney test.

Figure 9A shows the average distance of the observed generalization patterns to the above-mentioned strategies. The closest strategies to the observed generalization patterns are the similarity strategy and the filling pattern rule, followed by the random strategy, and finally by the shape rule. The two-sided Wilcoxon-Mann-Whitney test was performed to assess the difference in distribution between the different strategies. We found a significant difference between the shape rule and the random strategy ($p < .0001$), and between the random strategy and the filling pattern rule ($p < .0001$), but not between the filling pattern rule and the similarity strategy ($p = .16$). Significance values were adjusted using the Benjamini-Hochberg correction with a FDR $\le .05$. Since the shape rule and random strategy were the farthest to the observed patterns, they were excluded from the following analysis.

Figures 9B, C, D show the average distance of participants’ generalization patterns to two specific strategies (the filling pattern rule and the similarity strategy), as a function of the type of order within each manipulation (within-category in Fig. 9B, between-category in Fig. 9C, and across-blocks in Fig. 9D). While distances to the similarity strategy were similar across types of order within the same manipulation, distances to the filling pattern rule largely varied. More specifically, generalization patterns of participants in the rule-based order were significantly closer to the filling pattern rule than participant in the similarity-based order ($p = .034$). The same applies to participants in the interleaved study as compared to those in the blocked study ($p = .004$). Participants in the constant order were not significantly closer or farther from the filling pattern rule than those in the variable order ($p=.71$). With regard to the distance to the similarity strategy, none of the tests were significant ($p=.89$ for rule-based vs. similarity-based, $p=.16$ for blocked vs. interleaved, and $p=.15$ for constant vs. variable). An alternative analysis leading to similar results can be found in Supplementary material D.

**Figure 10:**
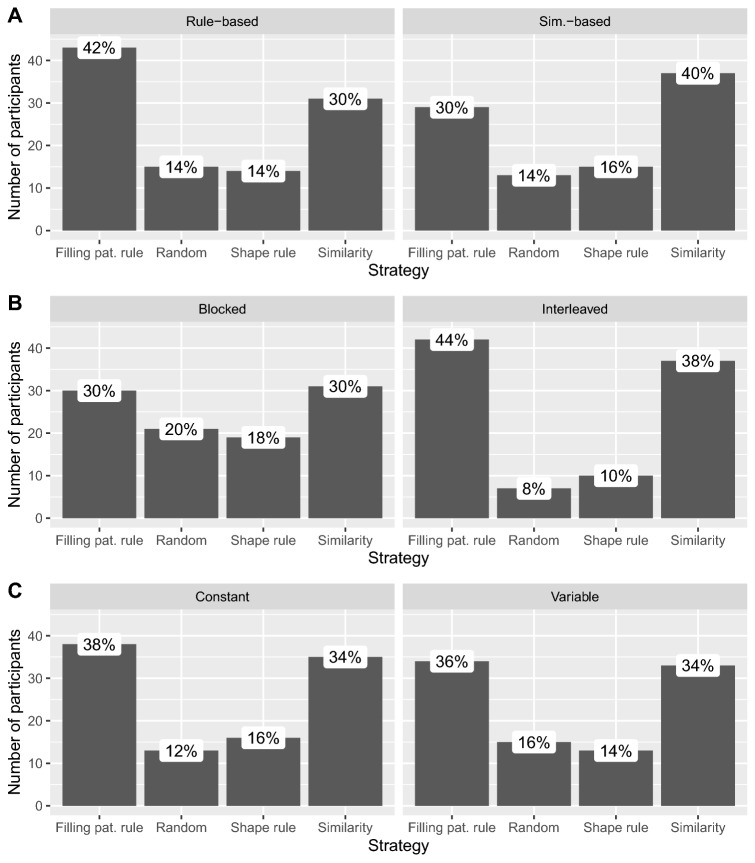
Partition of the participants in each main order type based on which strategy they most resemble. Graph (**A**) shows the within-category orders, graph (**B**) the between-category orders, and graph (**C**) the across-blocks manipulations. When there were multiple closest strategies, all of them were taken into account. The closest strategy was computed using the *L*1 norm.

To conduct a more thorough analysis of the transfer patterns, we partitioned the participants assigned to each type of order into groups, based on which strategy they best fit. For the partition, we considered all four strategies, and the best fit was determined by computing the *L*1 distance between the strategies and participants’ generalization patterns. The strategy associated with the minimum distance was the best fit. When there were multiple best fits, all of them were taken into account (nine participants reported two best fits, and two participants three). Figure 10 shows the result of this partition in terms of number and percentage of participants, for within-category (Fig. 10A), between-category (Fig. 10B), and across-blocks (Fig. 10C) orders. Participants in the rule-based order most often used the filling pattern rule (42%), followed by the similarity strategy (30%), and the two remaining strategies (14% each). Inversely, participants in the similarity-based order most often used the similarity strategy (40%), followed by the filling pattern rule (30%), the shape rule (16%), and the random strategy (14%). Participants in the blocked order most often used the filling pattern rule and the similarity strategy (30% each), followed by the random strategy (20%), and the shape rule (18%). In the interleaved order, only a very small percentage of participants used either the shape rule (10%) or the random strategy (8%), while the remaining subjects used either the filling pattern rule (44%) or the similarity strategy (38%). With regard to the across-blocks manipulations, the partition was similar across both types of order. The two-sided Fisher’s exact test of independence at level 0.05 found a significant difference in the partition of subjects between the blocked and interleaved orders ($p=.006$), but not between the rule-based and similarity-based orders ($p=.39$), nor between the constant and variable orders ($p=.93$).

## Discussion

Previous studies on category learning have shown that the sequence in which stimuli are encountered can profoundly influence learning speed and category formation^16,33,45^. However, the totality of these studies have only focused on a subset of the presentation orders (either within-category or between-category manipulations), ignoring potential interactions between different types of manipulations. Here, we manipulated within-category, between-category, and across-blocks order manipulations within a single task to study their separate and combined impact on learning speed and generalization patterns. Our main questions of interest are *(i)* whether manipulation of stimulus order across blocks influences how fast categories are learned, *(ii)* whether this type of manipulation interacts with other manipulations of order such as interleaving, blocking, grouping by rule, or grouping by similarity, and *(iii)* whether these experimental conditions facilitate the use of specific strategies on novel stimuli after the categories are learned.

To address the first question, we used two survival analysis techniques to analyze the time required by participants to reach the learning criterion, as a function of the conditions within each order manipulation. Both the Kaplan-Meier survival curves and Cox proportional-hazards model showed that the pace at which categories are learned is influenced by the across-blocks order. More specifically, the constant order was found to lead to faster learning than the variable order. Moreover, the analysis of subjects’ performance on learned stimuli during transfer showed better retention in the constant order as compared to the variable one.

To investigate whether the types of order interact in a more subtle fashion than what was revealed from the survival analyses, we performed a three-way ANCOVA with and without interactions. The between-subjects ANCOVA was used to analyze the interaction between within-category, between-category, and across-blocks orders on block-by-block performance after controlling for block number. Data was significantly best explained by the model with interactions, that found two significant interactions: one between within-category and across-blocks orders and the other between across-block and between-category orders. A further investigation of the interactions showed that the combination S+V (similarity-based + variable) is particularly detrimental for learning, whereas the combination B+C (blocked + constant) benefits leaning. This nuanced pattern also appears (at least for the combination S+V) in the Kaplan-Meier and Cox analyses with the eight experimental conditions (Fig. 3B and Fig. 4B). The ANCOVA also reported significant main effects for the three main manipulations. The fact that the ANCOVA reported a higher number of main effects than the survival analyses might be due to differences in the number of observations available (one learning time for the survival analyses vs. multiple block-by-block performance for ANCOVA).

To summarize, orders interact in a nuanced fashion that cannot be simply explained by aggregating the negative/positive effects of each type of order. The combination S+V amplified the detrimental effect of the variable order, making it the slowest condition. On the other hand, the combination B+C amplified the beneficial effect of the constant order. These nuanced interactions appear on top of the main effect of across-blocks manipulation, in which the constant order yields faster learning and better retention than the variable one.

The superiority of the constant across-blocks presentation over the variable one might be attributed to the limited amount of information carried by the constant order. Limiting the variability of the sequences might have helped participants focus on diagnostic information, enhancing the probability to either induct the simplest rule or memorize the category membership of the items. Another explanation is that memory can benefit from the repetition of the same sequences^48,49^, in particular to group items. A grouping process could have benefited the formation of rules.

In this study, we used the 5–4 category set that is often considered ‘rule-based’ in itself because of its use of discrete features. Therefore, our results most probably reflect the nature of this specific category structure. However, it is likely that more nuanced interactions should appear for other structures. For instance, an opposite effect could be hypothesized for information-integration category structures, where a rule based on binary decisions along dimensions is sub-optimal. For this type of structure, we might expect that the combination R+C would be the most detrimental, whereas the combination I+V would be the most beneficial. The bottom line is that order effects cannot be only analyzed in isolation, regardless of the category structure used in the task.

Our last set of analyses aimed at determining the influence of order manipulation on the classification of novel stimuli by analyzing the distance of the observed patterns to some main strategies. The rule-based strategy that uses Filling pattern as the main rule as well as the similarity-based strategy were found to be the closest to participants’ generalization patterns. Moreover, distance to the filling pattern rule was significantly higher for participants in the similarity-based order (as compared to those in the rule-based order) and for participants in the blocked order (as compared to those in the interleaved order). These results show that both within-category and between-category orders affect how learning is transferred to novel stimuli, with the filling pattern rule being preferred more often in the rule-based and interleaved conditions than in the similarity-based and blocked conditions, respectively.

The fact that the rule-based condition more clearly produces rule-based classification than the similarity-based condition has already been shown in^45^. Our results not only go in the same direction as in Mathy and Feldman, but also extend their findings to between-category orders. Moreover, our analysis relies on more nuanced transfer patterns than those used in this previous work. Indeed, instead of using whichever response was given more often in the five transfer blocks, we used the classification probability for each transfer stimulus.

Following the interpretation of Mathy and Feldman, the difference in strategy preference between rule-based and similarity-based learners might be attributed to the logic upon which the rule-based order is grounded. Presenting items following a “main rule (Filling pattern) plus exceptions” structure might have facilitated participants to infer the simplest rule, encouraging them to classify new items using the same inferred strategy. Conversely, the difference in preference between blocked and interleaved learners might find an explanation in the Sequential Attention Theory^72^. Alternating stimuli from different categories (interleaved study) leads to an attentional focus on properties that discriminate the categories, which might have promoted a rule-based transfer of the knowledge. By contrast, when stimuli from the same category are presented sequentially (blocked study), the encoding of the similarities among items of the same category is stronger, which might have promoted the use of a similarity-based strategy. In addition, the structure of the 5–4 category set (in particular the fact that the within-category similarity exceeds the between-category similarity) might also have played a role.

Finally, for a more thorough exploration of the transfer patterns, participants in each condition were partitioned based on the strategy they most resemble. Participants in the rule-based order preferred the filling pattern rule to the similarity strategy, whereas participants in the similarity-based order preferred the similarity strategy to the filling pattern rule. The blocked order promoted an almost equal use of the studied strategies, whereas the interleaved order almost exclusively promoted the use of the two most used strategies (i.e., the filling pattern rule and the similarity strategy).

An additional contribution of the present study is the promotion of underemployed statistical tools. A common practice in psychology is to remove participants who did not fulfill the objective of the task^45,73,74^. Nevertheless, unsuccessful participants can carry useful information. In the present study, we made use of two survival analysis techniques (the Kaplan-Meier survival curves and the Cox model) that allow us to account for individuals who did not complete the task. We advise the use of similar statistical tools when the conditions allow them.

### Study limitations and perspectives

Although Medin and Shaffer’s 5–4 category set has multiple benefits (see Introduction), it has the disadvantage of presenting a clear “rule plus exceptions” structure, which most likely benefited orders that facilitate rule acquisition. Future research should thus attempt to derive analogous results for other category structures. For instance, a potential perspective involves manipulating the same types of order in information-integration tasks to determine whether opposite effects stand.

Another limitation of this category structure is that it allows the co-existence of two different main rules that minimizes the number of exceptions (Filling pattern and Shape). Stimuli in the rule-based order were ordered following the “Filling pattern rule plus exceptions” structure. However, the dimension Shape could have equivalently been used instead of the dimension Filling pattern. In the same spirit, dimensions could have been instantiated by different features. For instance, Color could have distinguished the right and left objects within the cubes, Shape the objects at the front of the hypercube from those at the back, Size the objects at the top of the hypercube from those at the bottom, and Filling pattern the objects in the left cube from those in the right cube. We felt that multiplying our sample to consider all above-mentioned variations would have been too costly.

An additional perspective might involve using established computational models of category learning (such as SUSTAIN^75^) and recently developed ordinal models (such as the SAT-M^76^ or the OGCM^68^) to further explore the impact of various order combinations. Moreover, numerical simulations in the spirit of^77^ could be used as well to search for optimal order combinations.

## Supplementary Information

Supplementary Information.

## Data Availability

Data generated in the current study is available in Open Science Framework at https://osf.io/w29ts/?view_only=c28b965cc9a74c54b56d7adb87417ff1.

## References

[1] Bloom K, Shuell T (1981). Effects of massed and distributed practice on the learning and retention of second-language vocabulary. J. Educ. Res..

[2] Farrell S (2008). Multiple roles for time in short-term memory: Evidence from serial recall of order and timing. J. Exp. Psychol. Learn. Mem. Cogn..

[3] Wells GL (2014). Eyewitness identification: Probative value, criterion shifts, and policy regarding the sequential lineup. Curr. Dir. Psychol. Sci..

[4] Miller LM, Roodenrys S (2012). Serial recall, word frequency, and mixed lists: The influence of item arrangement. J. Exp. Psychol. Learn. Mem. Cogn..

[5] Helsdingen A, Gog T, Van Merrienboer JJG (2011). The effects of practice schedule on learning a complex judgment task. Learn. Instr..

[6] Kwan VSY, Wojcik SP, Miron-Shatz T, Votruba AM, Olivola CY (2012). Effects of symptom presentation order on perceived disease risk. Psychol. Sci..

[7] Jones M, Sieck W (2003). Learning myopia: An adaptive recency effect in category learning. J. Exp. Psychol. Learn. Mem. Cogn..

[8] Mack M, Palmeri T (2015). The dynamics of categorization: Unraveling rapid categorization. J. Exp. Psychol.: General.

[9] Mcdaniel M, Fadler C, Pashler H (2013). Effects of spaced versus massed raining in function learning. J. Exp. Psychol. Learn. Mem. Cogn..

[10] Sandhofer C, Doumas L (2008). Order of presentation effects in learning color categories. J. Cogn. Dev..

[11] Zeithamova D, Maddox W (2009). Learning mode and exemplar sequencing in unsupervised category learning. J. Exp. Psychol. Learn. Mem. Cogn..

[12] Zotov V, Jones M, Mewhort D (2011). Contrast and assimilation in categorization and exemplar production. Atten. Percept. Psychophys..

[13] Carvalho PF, Goldstone RL (2014). Effects of interleaved and blocked study on delayed test of category learning generalization. Front. Psychol..

[14] Carvalho PF, Goldstone RL (2014). Putting category learning in order: Category structure and temporal arrangement affect the benefit of interleaved over blocked study. Mem. Cogn..

[15] Carvalho PF, Goldstone RL (2015). The benefits of interleaved and blocked study: Different tasks benefit from different schedules of study. Psychon. Bull. Rev..

[16] Carvalho PF, Goldstone RL (2021). The most efficient sequence of study depends on the type of test. Appl. Cogn. Psychol..

[17] Goldstone RL (1996). Isolated and interrelated concepts. Mem. Cogn..

[18] Kornell N, Bjork R (2008). Learning concepts and categories: is spacing the“enemy of induction”?. Psychol. Sci..

[19] Kornell N, Castel A, Eich T, Bjork R (2010). Spacing as the friend of both memory and induction in young and older adults. Psychol. Aging.

[20] Kost, A. S., Carvalho, P. F. and Goldstone R. L. Can you repeat that? the effect of item repetition on interleaved and blocked study. *In Proceedings of the 37th Annual Meeting of the Cognitive Science Society. Austin, TX: Cognitive Science Society*, 1189–1194, 2015.

[21] Noh S, Yan V, Bjork R, Maddox W (2016). Optimal sequencing during category learning: Testing a dual-learning systems perspective. Cognition.

[22] Rohrer D (2009). The effects of spacing and mixing practice problems. J. Res. Math. Educ..

[23] Rohrer D (2012). Interleaving helps students distinguish among similar concepts. Educ. Psychol. Rev..

[24] Sana F, Yan V, Kim J (2016). Study sequence matters for the inductive learning of cognitive concepts. J. Educ. Psychol..

[25] Yan V, Soderstrom N, Seneviratna G, Bjotk E, Bjork R (2017). How should exemplars be sequenced in inductive learning? empirical evidence versus learners’ opinions. J. Exp. Psychol. Applied.

[26] Zulkiply N, Burt J (2012). The exemplar interleaving effect in inductive learning: Moderation by the difficulty of category discriminations. Mem. Cogn..

[27] Zulkiply N, Mclean J, Burt J, Bath D (2012). Spacing and induction: Application to exemplars presented as auditory and visual text. Learn. Instr..

[28] Carpenter SK, Cepeda NJ, Rohrer D, Kang SHK, Pashler H (2012). Using spacing to enhance diverse forms of learning: Review of recent research and implications for instruction. Educ. Psychol. Rev..

[29] Carpenter S, Mueller F (2013). The effects of interleaving versus blocking on foreign language pronunciation learning. Mem. Cogn..

[30] Cepeda NJ, Vul E, Rohrer D, Wixted JT, Pashler H (2008). Spacing effects in learning:A temporal ridgeline of optimal retention. Psychol. Sci..

[31] Hintzman DL, Summers JJ, Block RA (1975). What causes the spacing effect? some effects of repetition, duration, and spacing on memory for pictures. Mem. Cogn..

[32] Birnbaum M, Kornell N, Bjork E, Bjork R (2013). Why interleaving enhances inductive learning: The roles of discrimination and retrieval. Mem. Cogn..

[33] Kang S, Pashler H (2012). Learning painting styles: Spacing is advantageous when it promotes discriminative contrast. Appl. Cogn. Psychol..

[34] Wahlheim C, Finn B, Jacoby L (2012). Metacognitive judgments of repetition and variability effects in natural concept learning: Evidence for variability neglect. Mem. Cogn..

[35] Carvalho PF, Albuquerque PB (2012). Memory encoding of stimulus features in human perceptual learning. J. Cogn. Psychol..

[36] Carvalho, P. F. and Goldstone, R. L. Sequential similarity and comparison effects in category learning. *In Proceedings of the 33rd Annual Conference of the Cognitive Science Society*, 33:2977–2982, 07 2011.

[37] de Zilva D, Mitchell C (2012). Effects of exposure on discrimination of similar stimuli and on memory for their unique and common features. Q. J. Exp. Psychol..

[38] Rawson K, Thomas R, Jacoby L (2014). The power of examples: Illustrative examples enhance conceptual learning of declarative concepts. Educ. Psychol. Rev..

[39] Elio R, Anderson J (1981). The effects of category generalizations and instance similarity on schema abstraction. J. Exp. Psychol. Hum. Learn. Mem..

[40] Elio R, Anderson JR (1984). The effects of information order and learning mode on schema abstraction. Mem. Cogn..

[41] Bower G, Clark M, Lesgold A, Winzenz D (1969). Hierarchical retrieval schemes in recall of categorized word lists. J. Verbal Learn. Verbal Behav..

[42] Medin D, Bettger J (1994). Presentation order and recognition of categorically related examples. Psychon. Bull. Rev..

[43] Corcoran K, Epstude K, Damisch L, Mussweiler T (2011). Fast similarities: Efficiency advantages of similarity-focused comparisons. J. Exp. Psychol. Learn. Mem. Cogn..

[44] Mathy F, Feldman J (2009). A rule-based presentation order facilitates category learning. Psychon. Bull. Rev..

[45] Mathy F, Feldman J (2016). The influence of presentation order on category transfer. Exp. Psychol..

[46] Stewart N, Brown G, Chater N (2002). Sequence effects in categorization of simple perceptual stimuli. J. Exp. Psychol. Learn. Mem. Cogn..

[47] Sloman S (1996). The empirical case for two systems of reasoning. Psychol. Bull..

[48] French RM, Addyman C, Mareschal D (2011). TRACX: A recognition-based connectionist framework for sequence segmentation and chunk extraction. Psychol. Rev..

[49] O’Shea G, Clegg BA (2006). Stimulus and response chunking in the hebb digits task. Psychol. Res..

[50] Medin DL, Schaffer MM (1978). Context theory of classification learning. Psychol. Rev..

[51] Blair M, Homa D (2003). As easy to memorize as they are to classify: The 5–4 categories and the category advantage. Mem. Cogn..

[52] Cohen AL, Nosofsky RM (2003). An extension of the exemplar-based random-walk model to separable-dimension stimuli. J. Math. Psychol..

[53] Johansen M, Kruschke J (2005). Category representation for classification and feature inference. J. Exp. Psychol. Learn. Mem. Cogn..

[54] Johansen M, Palmeri T (2003). Are there representational shifts in category learning?. Cogn. Psychol..

[55] Lafond D, Lacouture Y, Mineau G (2007). Complexity minimization in rule-based category learning: Revising the catalog of boolean concepts and evidence for non-minimal rules. J. Math. Psychol..

[56] Lamberts K (2000). Information accumulation theory of categorization. Psychol. Rev..

[57] Minda J, Smith J (2002). Comparing prototype-based and exemplar-based accounts of category learning and attentional allocation. J. Exp. Psychol. Learn. Mem. Cogn..

[58] Rehder B, Hoffman A (2005). Thirty-something categorization results explained: Attention, eyetracking, and models of category learning. J. Exp. Psychol. Learn. Mem. Cogn..

[59] Smith J, Minda J (2000). Thirty categorization results in search of a model. J. Exp. Psychol. Learn. Mem. Cogn..

[60] Zaki SR, Nosofsky RM, Stanton RD, Cohen AL (2003). Prototype and exemplar accounts of category learning and attentional allocation: A reassessment. J. Exp. Psychol. Learn. Mem. Cogn..

[61] Nosofsky R, Kruschke J, McKinley S (1992). Combining exemplar-based category representations and connectionist learning rules. J. Exp. Psychol. Learn. Mem. Cogn..

[62] Nosofsky R, Gluck M, Palmeri T, Mckinley S, Glauthier P (1994). Comparing modes of rule-based classification learning: A replication and extension of shepard, hovland, and jenkins (1961). Mem. Cogn..

[63] Palmeri TJ, Nosofsky RM (1995). Recognition memory for exceptions to the category rule. J. Exp. Psychol. Learn. Mem. Cogn..

[64] Medin DL, Smith EE (1981). Strategies and classification learning. J. Exp. Psychol. Human Learn. Mem..

[65] Medin DL, Altom MW, Murphy TD (1984). Given versus induced category representations: Use of prototype and exemplar information in classification. J. Exp. Psychol. Learn. Mem. Cogn..

[66] Anderson JR, Betz J (2001). A hybrid model of categorization. Psychon. Bull. Rev..

[67] Faul F, Erdfelder E, Lang A-G, Buchner A (2007). G* power 3: A flexible statistical power analysis program for the social, behavioral, and biomedical sciences. Behav. Res. Methods.

[68] Mezzadri G, Reynaud-Bouret P, Laloë T, Mathy F (2022). An order-dependent transfer model in categorization. J. Math. Psychol..

[69] Kaplan EL, Meier P (1958). Nonparametric estimation from incomplete observations. J. Am. Stat. Assoc..

[70] Cox DR (1972). Regression models and life-tables. J. R. Stat. Soc. Ser. B (Methodological).

[71] Cramer AO, van Ravenzwaaij D, Matzke D, Steingroever H, Wetzels R, Grasman RP, Waldorp LJ, Wagenmakers E-J (2016). Hidden multiplicity in exploratory multiway anova: Prevalence and remedies. Psychon. Bull. Rev..

[72] Carvalho PF, Goldstone RL (2015). What you learn is more than what you see: What can sequencing effects tell us about inductive category learning?. Front. Psychol..

[73] Meagher B, Carvalho P, Goldstone R, Nosofsky R (2017). Organized simultaneous displays facilitate learning of complex natural science categories. Psychon. Bull. Rev..

[74] Meagher B, Cataldo K, Douglas B, Mcdaniel M, Nosofsky R (2018). Training of rock classifications: The use of computer images versus physical rock samples. J. Geosci. Educ..

[75] Love B, Medin D, Gureckis T (2004). Sustain: A network model of category learning. Psychol. Rev..

[76] Carvalho PF, Goldstone RL (2022). A computational model of context-dependent encodings during category learning. Cogn. Sci..

[77] Nosofsky RM, Sanders C, Zhu X, Mcdaniel M (2018). Model-guided search for optimal natural-science-category training exemplars: A work in progress. Psychon. Bull. Rev..

