# Supplementary material for: Investigating interactions between types of order in categorization
Source: Sci Rep. 2022 Dec 14;12:21625. doi: 10.1038/s41598-022-25776-0 (PMC9751307; doi:10.1038/s41598-022-25776-0)
Supplement: Supplementary file 1 — Supplementary Information. [file 41598_2022_25776_MOESM1_ESM.pdf]

Supplementary material

A Analysis of the number of unsuccessful participants

The term "unsuccessful participants" refers to those individuals who did not meet the learning criterion, either because they dropped out or because they exceeded the maximal amount of blocks allowed to the experiment (200 learning blocks). Table S1 shows the number of successful and unsuccessful participants per types of order, taken both separately and combined. The two-sided Fisher's exact test of independence at 0.05 level was run to determine whether the number of individuals who did not reach the learning criterion was related to the type of order. The Fisher's exact test was preferred to the chi-square test because of its accuracy with small samples. None of the tests were significant ($p = .46$ for rule-based vs. similarity-based, $p = .46$ for blocked vs. interleaved, $p = 0.81$ for constant vs. variable, and $p = .06$ for the omnibus test run on the table showing all conditions). We conclude that the type of order in which stimuli were encountered did not significantly alter participants' chance of reaching the learning criterion.

B Analysis of the percentage of correct responses during learning

Because we were interested in analyzing the learning curves of participants who learned the studied categories, unsuccessful participants (amounting to eighteen) were excluded from the analysis. Figure S1 shows the average percentage of correct responses among participants within a same type of order taken separately (Figure S1A) and combined (Figure S1B), as a function of block number over the course of the learning phase. Only participants' performance during no-feedback blocks is represented in the graph. Because it was reasonable to think that successful participants would have continued to correctly classify stimuli after reaching the learning criterion, their responses were completed with the highest performance (i.e., 100% of correct responses) until block number 63. Because 80% of the participants ended the learning phase before block number 63, this choice appeared to be a good trade-off between minimizing the number of observations that were both removed and added. In Figure S1A, learning was more efficient in the rule-based and constant orders than in the similarity-based and variable orders, respectively. To assess the difference between the learning curves, we performed the two-sided Wilcoxon-Mann-Whitney test at each block with Benjamini-Hochberg adjustment for multiple testing corrections. One third of the tests were significant in the within-category order (7/21 rejected tests), none of the tests were significant in the between-category order, and only one test was significant in the across-blocks manipulations. Although visually both rule-based and constant orders appeared to benefit learning, we only found evidence supporting a more effective learning in the rule-based order as compared to the similarity-based one.

C Alternative way of filling in the data in the three-way ANCOVA

In this section, we use an alternative way of filling in the data, and run the three-way ANCOVA a second time. We here filled in the data by repeating participants' average performance on the last two no-feedback blocks (instead of using the last no-feedback block). Block number was a significant predictor of participants' performance ($F(1, 3305) = 1005.74$, $p < .0001$, $\eta_p^2 = .182$). The main effect of within-category order was significant ($F(1, 271) = 82.55$, $p < .0001$, $\eta_p^2 = .018$), as well as the one of between-category order ($F(1, 60) = 18.12$, $p < .0001$, $\eta_p^2 = .004$) and the one of across-blocks order ($F(1, 83) = 25.17$, $p < .0001$, $\eta_p^2 = .006$). With regard to the interactions, we found a significant interaction between within-category and across-blocks orders ($F(1, 16) = 4.95$, $p = .03$, $\eta_p^2 = .001$), and between between-category and across-blocks orders ($F(1, 18) = 5.59$, $p = .02$, $\eta_p^2 = .001$). No significant interaction was found between within-category and between-category orders ($F(1, 2) = 0.66$, $p = .4$, $\eta_p^2 < .001$). Finally, the interaction between the three types of order was significant ($F(1, 42) = 12.83$, $p = .0005$, $\eta_p^2 = .003$). All p-values were corrected for multiple comparisons using the Benjamini-Hochberg correction with a FDR ≤ 0.05.

D Alternative analysis of generalization patterns on transfer stimuli

The aim of this analysis is to determine whether participants in different conditions classify new items using different strategies. We focus on two main strategies: a rule-based strategy that uses Filling pattern as main rule and a similarity-based strategy (see Figure 8 in the manuscript). A learner adopting the filling pattern rule would more often classify items T_1 and T_2 into category A and items T_4, T_5 and T_6 into category B, than a learner adopting the similarity strategy. Therefore, these two strategies can be distinguished by projecting generalization

patterns on the one-dimensional space generated by vector $v = (1, 1, 0, -1, -1, -1, 0)$. The more the projections are on the right side of this one-dimensional space, the more the filling pattern rule was used. Figure S2 shows the projection value of participants' generalization patterns on the one-dimensional space generated by vector v as a function of the conditions within each order manipulation. The two-sided Wilcoxon-Mann-Whitney test was performed to assess the difference between projection values of participants in different conditions. Projections from participants in the rule-based order were significantly higher than those from participants in the similarity-based order ($p = .028$). Similarly, projections from participants in the interleaved order were significantly higher than those from participants in the blocked order ($p = .010$). No significant difference was found between projections from participants with different across-blocks orders ($p = .86$). We conclude that participants in the rule-based and interleaved orders showed generalization patterns that where more consistent with the filling pattern rule than participants in the similarity-based and blocked orders, respectively.

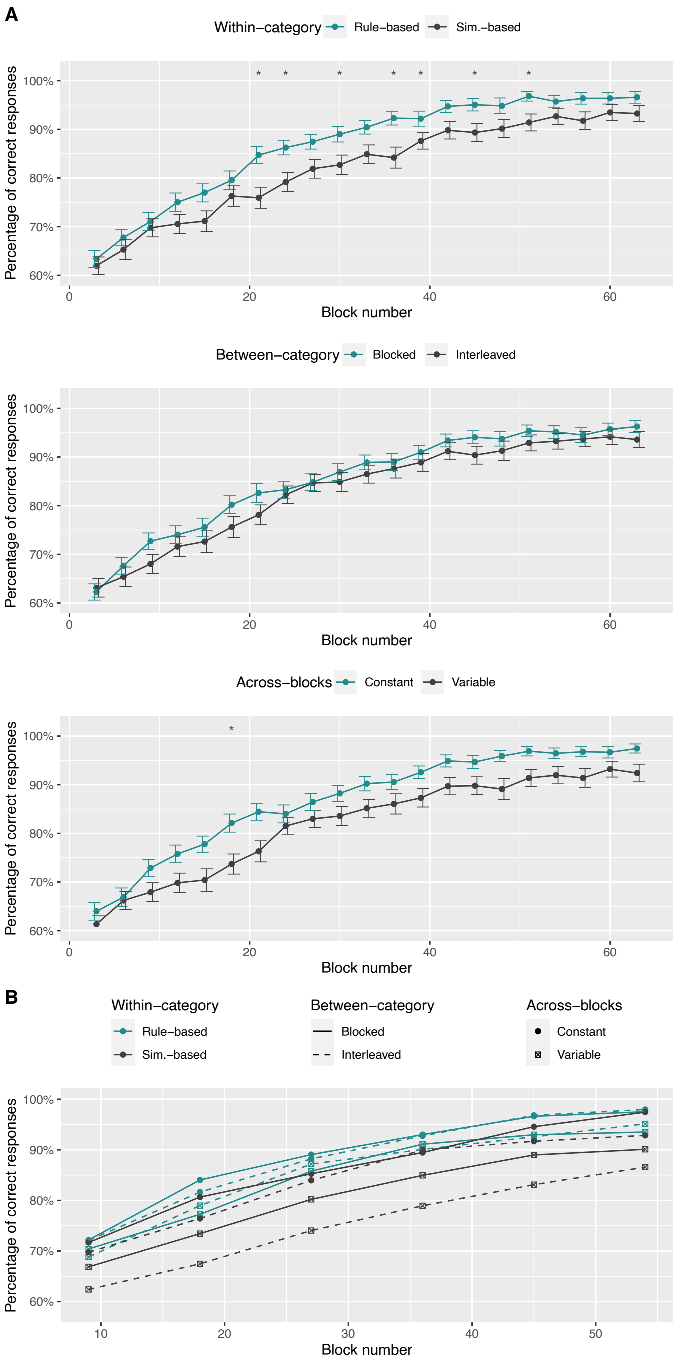

Figure S1: Average percentage of correct responses among participants within a same type of order taken separately (Figure A) and combined (Figure B), as a function of block number over the course of the learning phase. In Figure A, only performance across no-feedback blocks are plotted. Asterisks indicate the blocks on which the Wilcoxon-Mann-Whitney test with the Benjamini-Hochberg correction was rejected. Error bars show $\pm 1SE$. In Figure B, color distinguishes the within-category order (blue for rule-based and gray for similarity-based), line-type distinguishes the between-category order (solid line for blocked and dashed line for interleaved), and shape distinguishes the across-blocks order (dots for constant and crossed squares for variable). To increase smoothness of the curves, performance at each no-feedback block are obtained by averaging performance from the two preceding no-feedback blocks, the current no-feedback block, and the two following no-feedback blocks. To make the plot more readable, only performance every three no-feedback blocks are plotted.

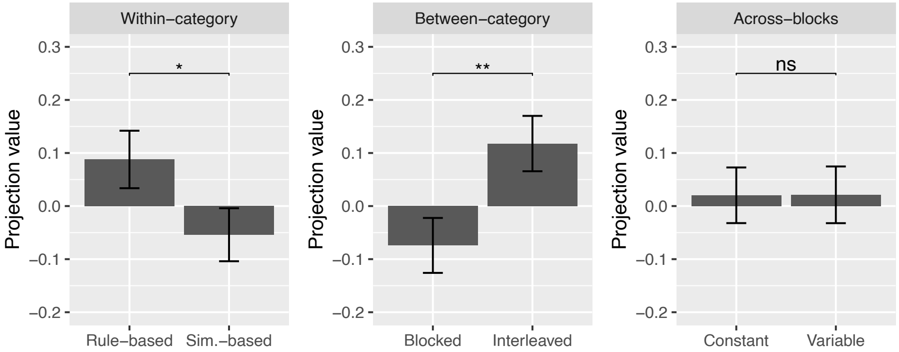

Figure S2: Projection value of the observed generalization patterns, as a function of different conditions within each order manipulation. Generalization patterns were projected on the one-dimensional space generated by vector $v = (1, 1, 0, -1, -1, -1, 0)$, which distinguishes the filling pattern rule-based strategy from the similarity-based strategy. Error bars show $\pm 1SE$. Asterisks show the significance of the two-sided Wilcoxon-Mann-Whitney test.

Type of order	Successful	Unsuccessful
Within-category orders		
Rule-based	101	7
Similarity-based	97	11
Between-category orders		
Blocked	101	7
Interleaved	97	11
Across-blocks orders		
Constant	100	8
Variable	98	10
Conditions		
R + B + C	27	0
R + B + V	25	2
R + I + C	23	4
R + I + V	26	1
S + B + C	27	0
S + B + V	22	5
S + I + C	23	4
S + I + V	25	2

Table S1: Number of successful and unsuccessful participants in each type of order, taken both separately and combined. Note that the total is 216 participants.
